# PK/PD of Positively Charged ADC in Mice

**DOI:** 10.3390/pharmaceutics17030377

**Published:** 2025-03-17

**Authors:** Hsuan-Ping Chang, Huyen Khanh Le, Shufang Liu, Dhaval K. Shah

**Affiliations:** Department of Pharmaceutical Sciences, School of Pharmacy and Pharmaceutical Sciences, The State University of New York at Buffalo, Buffalo, NY 14214, USA; hsuanpin@buffalo.edu (H.-P.C.); huyenle@buffalo.edu (H.K.L.);

**Keywords:** ADC, positive charge, pharmacokinetics, liver, spleen

## Abstract

**Background/Objectives**: Antibody–drug conjugates (ADCs) show significant promise in oncology but often suffer from a narrow therapeutic window. Introducing a positive charge on the antibody is one proposed strategy to enhance tumor distribution and efficacy of ADC. Accordingly, this study evaluates the pharmacokinetics (PK) and pharmacology of an ADC developed using a positively charged (+5) version of anti-HER2 antibody trastuzumab conjugated with vc-MMAE linker-payload. **Methods**: A positively charged variant of trastuzumab was generated and conjugated to vc-MMAE. In vitro cytotoxicity assays were performed in cell lines with varying HER2 expression levels: N87 (high), MCF-7 (low), and MDA-MB-468 (non-expressing). In vivo biodistribution of wild-type (WT) and positively charged (+5) ADC was investigated in plasma, tumors, liver, and spleen. A pilot efficacy and toxicity study was also conducted in N87 tumor-bearing mice. **Results**: The charged ADC showed differential potency and PK behavior compared to the WT ADC. The charged ADC had similar potency in N87 cells but demonstrated ~20-fold and ~60-fold higher potency in MCF-7 and MDA-MB-468 cells. Plasma exposures of all the analytes were found to be reduced following the administration of charged ADC. However, total antibody exposure was found to increase in liver, spleen, and low antigen-expressing MCF-7 tumors. Tumor payload exposures were found to be significantly reduced for the charged ADCs, but liver and spleen displayed higher peak concentrations and increased tissue-to-plasma exposure ratios for the payload, suggesting preferential distribution of ADC with high drug–antibody ratio (DAR) to liver and spleen. Consistent with reduced tumor exposures, charged ADC showed lower efficacy in N87 tumor-bearing mice. No overt toxicity was observed for the charged ADC. **Conclusions**: Our findings suggest that while positively charged ADCs may be more potent in vitro, their efficacy in vivo may be compromised due to altered PK behavior. Thus, introducing a positive charge into the antibody framework may not be a viable strategy for improving the therapeutic potential of ADCs.

## 1. Introduction

Antibody–drug conjugates (ADCs) have emerged as an important class of targeted therapeutics in oncology, which leverages the specificity of monoclonal antibodies (mAbs) to deliver potent cytotoxic agents directly into the tumor cells [1]. As of 2025, 14 ADCs have been approved by the FDA, with numerous other ADCs undergoing clinical trials [2]. Despite their therapeutic potential, the clinical utility of ADCs is currently hindered by a relatively narrow therapeutic window, which limits their widespread clinical applications [3]. Various strategies have been explored to improve the therapeutic window of ADCs, such as engineering the targeting modality, changes in linker chemistry, and alteration in payloads [4]. However, the effect of the physicochemical properties of antibodies, especially charge, on ADC pharmacology has not been explored in detail. Therefore, in this manuscript, we have investigated how the charge of an antibody affects the pharmacokinetics (PK) and pharmacodynamics (PD) of an ADC in mice.

Antibody charge has been shown to significantly impact the biodistribution of antibodies [5,6], which can potentially alter the PK profile of ADC as well. It has been suggested that a positive charge can promote mAb interaction with the negatively charged cell membrane and extracellular matrix (ECM), resulting in enhanced pinocytosis and cellular uptake of mAb [7]. Notably, studies have shown that increasing the positive charge of antibodies can improve mAb tumor distribution, where increased tissue-to-plasma and interstitial fluid (ISF)-to-plasma exposure ratios for tumors were associated with the positive charge of antibodies [8]. Considering the potential of positively charged mAb in increasing tumor exposure, we hypothesize that positively charged antibody-based ADC may show elevated tumor uptake and ultimately improved ADC efficacy.

To investigate this hypothesis, we developed an ADC using a positively charged variant of the anti-human HER2 antibody trastuzumab and valine-citrulline-monomethyl auristatin E (vc-MMAE) linker-payload. We evaluated the in vitro cytotoxicity of the ADC in various cancer cell lines with different HER2 expression levels to determine the effect of charge on ADC potency in vitro. Given that nonspecific pinocytosis may also increase the risk of the ADC entering normal tissues and potentially lead to off-target effects, we also conducted PK studies with the positively charged ADC in xenograft tumor-bearing mice and examined the biodistribution of ADC in plasma, tumors, and selected tissues. ADC analyte concentrations, encompassing both the antibody and payload components, were determined using ligand-binding assay and LC–MS/MS–based assay, respectively. Pilot efficacy studies were also conducted in the mouse model to evaluate the effect of charge on ADC efficacy in vivo. Collectively, the investigation conducted here was designed to help elucidate the effect of antibody charge on the therapeutic potential of ADCs.

## 2. Materials and Methods

### 2.1. Production and Characterization of Wild-Type (WT) and Positively Charged mAb

Wild-type (WT) and positively charged variants of the anti-HER2 antibody trastuzumab were produced in-house. For acquiring the positively charged antibody, the surface-exposed residues on the complementary determining region were mutated to positively charged lysine or arginine, resulting in an antibody variant possessing positive charge patches on the variable domain with a net charge of +5. More details about the design of the positively charged variant have been published before [7]. Variable light (VL), variable heavy (VH), and constant heavy (CH) gene variants for antibodies were synthesized and provided by Synbio Technologies (Monmouth Junction, NJ, USA) and subcloned into an in-house IgK-FRT expression vector. The resulting plasmids were transfected into Chinese Hamster Ovary (CHO) cells using Lipofectamine™ 3000 (Cat# L3000015, Thermo Fisher Scientific, Grand Island, NY, USA). Antibodies secreted into the medium were collected and purified on a HiTrap™ Protein G HP column (Cat# 17-0405-01, GE Healthcare, Chicago, IL, USA). The column was equilibrated with 20 mM phosphate buffer (pH 7), and bound mAbs were eluted with glycine buffer (pH 2.7), then buffer-exchanged into PBS. Purified mAbs were stored at −20 °C. The identity and purity of both the WT and positively charged mAbs were confirmed by SDS-PAGE. The binding affinity to HER2 for the positively charged mAb was confirmed by a previous study [9], which demonstrated that charge modifications in mAb did not notably impact its binding to the target.

### 2.2. Synthesis and Characterization of WT and Positively Charged ADCs

WT and positively charged ADCs were synthesized by conjugating each antibody to a vc-MMAE linker-payload using a previously established random conjugation method [10]. Briefly, partially reduced interchain disulfide bonds were generated by incubation with tris(2-carboxyethyl)phosphine (TCEP), followed by a reaction with around eight molar equivalents of vc-MMAE (maleimidocaproyl-Val-Cit-MMAE). After conjugation, the reaction mixture was purified on a Sephadex G-25 column to remove excess linker payload. The drug–antibody ratio (DAR) of WT ADC was determined by hydrophobic interaction chromatography (HIC) [11]. Briefly, mobile phase A consisted of 1.5 M ammonium sulfate in 50 mM phosphate buffer (pH 7), while mobile phase B contained 50 mM phosphate buffer with 20% isopropanol at the same pH. ADCs were run for 18 min at a flow rate of 0.8 mL/min, and peak area integration of each respective drug payload was performed to calculate the overall DAR value. While the HIC method was optimized for wild-type ADC, it was not optimized for the positively charged ADC due to potential alterations in retention characteristics. Therefore, an alternative forced deconjugation method was employed to evaluate conjugated MMAE. To estimate the average DAR of the positively charged ADC, the DAR of WT ADC characterized by HIC was used as a reference. A range of concentrations (200 to 2000 nM) of WT ADC and positively charged ADC were spiked into plasma and incubated with cysteine protease papain (Sigma-Aldrich, St. Louis, MO, USA) for 8 h, allowing complete deconjugation of MMAE from the antibody. The total released MMAE concentration was then quantified using LC–MS/MS, and the ratios of MMAE to the internal standard (IS) were recorded. The difference in MMAE/IS ratios between WT ADC and the positively charged ADC was used to estimate the average DAR for the positively charged ADC.

### 2.3. In Vitro Cytotoxicity of Positively Charged ADC

The MTT (3-(4, 5-dimethylthiazol-2-yl)-2,5-diphenyltetrazolium bromide) assays were performed using WT ADC and positively charged ADC in three cell lines with varying HER2 expression levels: N87 (high), MCF-7 (low), and MDA-MB-468 (non-expressing). Cells were seeded at a density of 10,000 cells per well in 96-well plates and incubated for 24 h before treatment. ADCs were added at concentrations ranging from 0.01 nM to 1000 nM, and cells were incubated for an additional 96 h. Following treatment, 5 mg/mL MTT solution was added to each well and incubated for 4 h, after which 10% SDS-HCl solution was added to dissolve the formazan crystals. Absorbance was measured at 570 nm the next day. Cell viability (%) was calculated as (OD_treated_/(OD_control_) × 100%. GraphPad Prism (version 10.4.1) was used for curve fitting of the sigmoidal dose-response curves (cell viability vs. ADC concentration) under the nonlinear regression option, and the half-maximal inhibitory concentration (IC_50_) values were determined.

### 2.4. In Vivo Biodistribution Study of Positively Charged ADC

#### 2.4.1. Development of Xenograft Mouse Model

The gastric carcinoma cell line NCI-N87 (CRL-5822™) and breast cancer cell line MCF-7 were obtained from the American Type Culture Collection (ATCC, Manassas, VA, USA) and used to establish xenograft tumors. Cells were cultured in RPMI 1640 medium (ATCC^®^ 302001™) supplemented with 10% (*v/v*) heat-inactivated fetal bovine serum (FBS) (Gibco/Thermo Fisher Scientific, Grand Island, NY, USA) and 10 μg/mL gentamicin (Sigma-Aldrich, St. Louis, MO, USA) in a humidified incubator at 37 °C with 5% CO_2_.

Male athymic nude mice (IMSR_JAX:007850) were purchased at five weeks of age from The Jackson Laboratory (Bar Harbor, ME, USA). Following a two-week acclimation period, mice were subcutaneously inoculated with either NCI-N87 or MCF-7 cells in the right dorsal flank. All in vivo procedures adhered to the Principles of Laboratory Animal Care (National Institutes of Health publication 85–23, revised 1985) and were approved by the University at Buffalo Institutional Animal Care and Use Committee (IACUC#PROTO202100089).

#### 2.4.2. Pharmacokinetics of Positively Charged ADC in Plasma, Tumor, and Tissues

A total of 60 outbred homozygous male athymic nude mice were used for PK studies. Thirty mice were implanted with NCI-N87 tumors and 30 with MCF-7 tumors. For each xenograft model, 15 mice received WT ADC (10 mg/kg), and 15 received positively charged ADC (10 mg/kg), both administered intravenously. Groups of three mice were sacrificed at 10 min, 6 h, 24 h, 72 h, and 168 h post-dose. Blood, tumors, and tissue samples, including liver and spleen, were collected to quantify ADC analytes: total mAb (sum of conjugated and unconjugated mAbs), total MMAE (sum of conjugated and unconjugated MMAE), and unconjugated MMAE. Following quantification, non-compartmental analysis (NCA) was conducted on the PK data using WinNonlin (version 8.4, Pharsight, Mountain View, CA, USA).

### 2.5. Bioanalytical Methods

#### 2.5.1. Sample Pretreatment

Tumor and tissue homogenization was performed as previously described [12]. Briefly, radioimmunoprecipitation assay (RIPA) buffer containing a protease inhibitor was added to weighed tumor and tissue samples at a dilution factor of 5. Samples were homogenized using a BeadBug™ microtube homogenizer (Benchmark Scientific, Sayreville, NJ, USA) at maximum speed for 15 s, followed by a 30 s cooling period on ice. This process was repeated 3–5 times until complete homogenization was achieved.

#### 2.5.2. ELISA for Total mAb Quantification

Total mAb concentrations were measured using a validated sandwich ELISA for plasma, tumor, and tissue samples [12]. Briefly, 384 well plates were coated with anti-human IgG-F(ab’)2 monoclonal antibody overnight at 4 °C, followed by a 1-h blocking step at room temperature. Samples, standards, and quality controls (QCs) were added and incubated for 2 h. Goat anti-human IgG-F(ab’)2 conjugated with alkaline phosphatase was used as the detection antibody. The positively charged ADC was used to make the standards and QCs for positively charged ADC quantification, and WT ADC was used to make the standards and QCs for WT ADC quantification. Absorbance at 405 nm was monitored over time, and all standard curves were fitted using a four-parameter logistic equation.

#### 2.5.3. LC–MS/MS for Unconjugated MMAE Quantification

Unconjugated MMAE was quantified using a Shimadzu 8040 LC–MS/MS system with electrospray ionization and triple quadrupole mass spectrometry. Chromatographic separation was performed on an XBridge BEH Amide column (Waters Corporation, Milford, MA, USA) using water as the aqueous phase and 95:5 acetonitrile/water (both containing 5 mM ammonium formate and 0.1% formic acid) as the organic phase. The chromatographic run time was 12 min, with multiple reaction monitoring (MRM) transitions at *m*/*z* 718.5→686.5 and *m*/*z* 718.5→152.1.

Deuterated MMAE (d8-MMAE; MCE MedChem Express, Monmouth Junction, NJ, USA) was used as an internal standard. Samples (standards, QCs, plasma, tumor, and tissue homogenates) were spiked with d8-MMAE, followed by protein precipitation with acetonitrile. After vortexing and centrifugation at 15,000× *g* for 15 min at 4 °C, the supernatants were collected, evaporated, and reconstituted in 50 µL of 95:5 acetonitrile/water (0.1% formic acid) before LC–MS/MS analysis [13].

#### 2.5.4. Papain Deconjugation for Total MMAE Quantification

Total MMAE was quantified using a papain-based deconjugation method [14]. Plasma, tumor, and tissue samples were incubated with cysteine protease papain (Sigma-Aldrich, St. Louis, MO, USA) at 37 °C for 8 h to release MMAE from the ADC. Following incubation, samples were processed as described in the LC–MS/MS for unconjugated MMAE section for total MMAE quantification.

### 2.6. In Vivo Efficacy Study and Toxicity Assessment of Positively Charged ADC

For the efficacy study, 13 male athymic nude mice bearing NCI-N87 tumors were randomized into three groups. WT ADC and positively charged ADC (average DAR of ~3.0) were administered intravenously at 5 mg/kg dose to two groups of five mice each, while the remaining three mice received PBS as a control. Tumor dimensions (length and width) were measured using a vernier caliper every other day for up to 60 days or until tumor volume exceeded 2000 mm^3^. Tumor volume (V) was calculated as ½ × length × width^2^. Body weight was also monitored every other day for 60 days as an indicator of systemic toxicity.

## 3. Results

### 3.1. Synthesis and Characterization of Positively Charged ADCs

The purity and structural integrity of WT and positively charged mAbs were confirmed by reducing and non-reducing SDS-PAGE (Figure S1). The previously established conjugation protocol for WT vc-MMAE ADC was further optimized for the positively charged ADC, requiring approximately 3 molar equivalents of tris(2-carboxyethyl)phosphine (TCEP) for WT ADC and ~4 molar equivalents of TCEP for the positively charged ADC as the reducing agent. The DAR was confirmed to be approximately 4 for WT ADC using HIC (Figure S2). Since HIC was not optimized for the positively charged ADC, the forced deconjugation method was used to estimate its DAR. The ratios of MMAE/IS between the positively charged ADC and WT ADC were found to be 70% across the range of ADC concentrations tested, suggesting a DAR of approximately 3 for the positively charged ADC.

### 3.2. In Vitro Cytotoxicity of Positively Charged ADC

**Figure 1:**
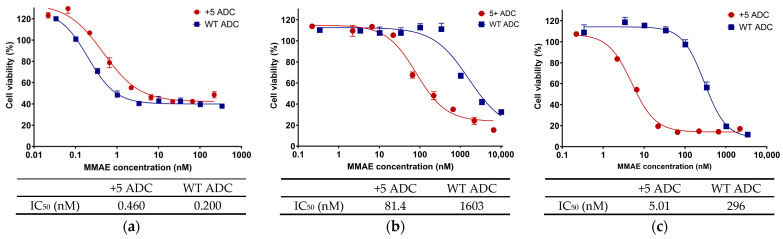
In vitro cytotoxicity of WT and positively charged (+5) ADCs in (**a**) HER2 high-expressed NCI-N87 cells, (**b**) HER2 low-expressed MCF-7, and (**c**) HER2 non-expressed MDA-MB 468 cells.

Cytotoxicity was evaluated via MTT assays for both WT ADC and positively charged ADC in NCI-N87 (HER2 high-expressing; Figure 1a), MCF-7 (HER2 low-expressing; Figure 1b), and MDA-MB-468 (HER2 non-expressing; Figure 1c) cell lines, with the corresponding IC_50_ values presented below each figure panel. In NCI-N87 cells, both ADCs exhibited sub-nanomolar IC_50_ values, although the positively charged ADC showed slightly lower potency, with IC_50_ values within a 2–3-fold range compared to the WT ADC (Figure 1a), potentially reflecting variations in DAR between the two formulations. By contrast, in MCF-7 and MDA-MB-468 cells, the positively charged ADC demonstrated markedly higher potency, with IC_50_ values ~20-fold and ~60-fold lower than the WT ADC, respectively (Figure 1b,c). This enhanced potency in vitro may be attributed to increased pinocytosis of the charged antibody, as observed before [7], allowing for more effective intracellular delivery of the ADC payload in cells with low or absent HER2 expression.

### 3.3. PK of Positively Charged ADC in Plasma

**Figure 2:**
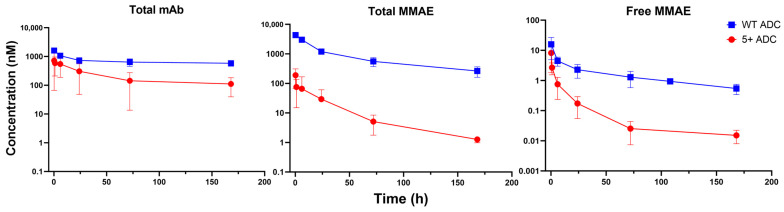
Plasma PK of total mAb, total MMAE, and unconjugated MMAE following WT and positively charged (+5) ADC administration in mice.

Figure 2 depicts the plasma PK profiles of total mAb, total MMAE, and unconjugated MMAE for both WT and positively charged ADCs. The PK parameters derived from NCA are summarized in Table 1. Compared with WT ADC, the positively charged ADC exhibited a 9-fold higher clearance and a 5-fold shorter half-life for total mAb. Additionally, it displayed a 2-fold larger volume of distribution, suggesting enhanced tissue distribution. As a result, total mAb exposure for the positively charged ADC was significantly lower than that of WT ADC (Table 1). Regarding total MMAE, the positively charged ADC showed substantially reduced exposure in terms of both peak concentration ($C_{max}$) and area under the plasma concentration-time curve (AUC) compared to WT ADC (Table 1), with these differences observed within the first 10 min post-administration (Figure 2). For unconjugated MMAE, the positively charged ADC displayed a lower AUC and a 4-fold shorter half-life relative to WT ADC, although their $C_{max}$ were similar (Table 1). Additionally, unconjugated MMAE accounted for approximately 1% of the total MMAE systemic exposure in the positively charged ADC group and 0.2% in the WT ADC group (Table 1).

### 3.4. PK of Positively Charged ADC in Tumors

**Figure 3:**
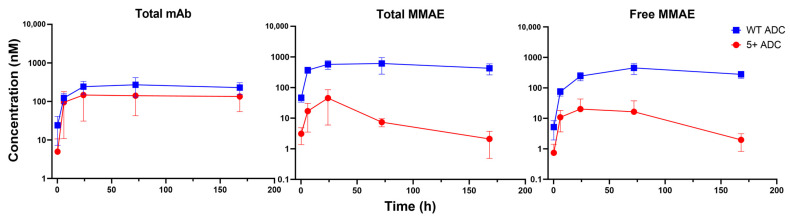
PK of total mAb, total MMAE, and unconjugated MMAE for WT and positively charged (+5) ADC in NCI-N87 tumors.

**Figure 4:**
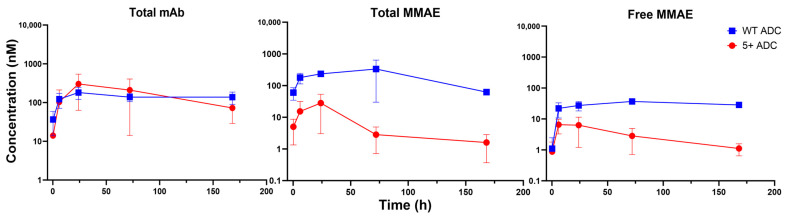
PK of total mAb, total MMAE, and unconjugated MMAE for WT and positively charged (+5) ADC in MCF-7 tumors.

Figure 3 and Figure 4 illustrate the PK profiles of various ADC analytes for WT and positively charged ADCs in NCI-N87 tumors (high HER2 expression) and MCF-7 tumors (low HER2 expression), respectively. The PK parameters derived from NCA for NCI-N87 and MCF-7 tumors are provided in Table 2 and Table 3, respectively.

In NCI-N87 tumors, total mAb PK was generally comparable between the positively charged and WT ADCs, with the positively charged ADC showing a slightly lower AUC than the WT ADC. In contrast, in MCF-7 tumors, the positively charged ADC displayed a ~2-fold higher $C_{max}$ but a similar AUC compared to the WT ADC, suggesting that charge-driven distribution may affect tumor uptake in low HER2-expressing tumors.

Despite having comparable total mAb exposure in both tumor models, the positively charged ADC exhibited significantly lower total and unconjugated MMAE exposure ($C_{max}$ and AUC) relative to the WT ADC. Specifically, in NCI-N87 tumors, total MMAE AUC was 40-fold lower, and unconjugated MMAE AUC was 30-fold lower for the positively charged ADC. In MCF-7 tumors, total MMAE AUC was 30-fold lower, and unconjugated MMAE AUC was 10-fold lower. These findings suggest that the positively charged ADC may not enhance drug payload delivery to tumors. Considering that ADC formulations contain multiple DAR species, we hypothesize that lower DAR species of the positively charged ADC may preferentially localize to tumors, while higher DAR species could accumulate more extensively in normal tissues, as discussed in the following section.

### 3.5. PK of Positively Charged ADC in Tissues

**Figure 5:**
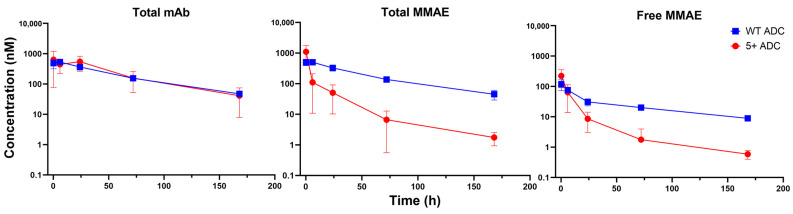
PK of total mAb, total MMAE, and unconjugated MMAE for WT and positively charged (+5) ADC in the liver.

The PK of different ADC analytes for WT and positively charged ADCs were also evaluated in the liver and spleen. Figure 5 shows the PK profiles in the liver, with parameters derived by NCA presented in Table 4. For total mAb, the PK profile of the positively charged ADC was comparable to that of the WT ADC. However, payload exposure in the liver differed significantly, with total and unconjugated MMAE exposure (AUC) for the positively charged ADC approximately 3–5-fold lower than that of the WT ADC. At the first time points where $C_{max}$ was reached, on the other hand, concentrations of both total and unconjugated MMAE in the liver were 2-fold higher for the positively charged ADC. This observation aligns with previous findings suggesting that high-DAR species of positively charged ADCs can accumulate in tissues early. Because MMAE conjugation increases the overall hydrophobicity, these results indicate that both charge and hydrophobicity affect ADC tissue distribution.

**Figure 6:**
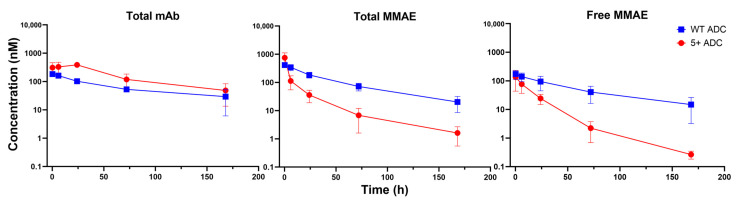
PK of total mAb, total MMAE, and unconjugated MMAE for WT and positively charged (+5) ADC in the spleen.

Figure 6 presents the PK profiles of ADC analytes in the spleen, with PK parameters summarized in Table 5. Unlike the liver, the spleen showed higher total mAb concentrations of the positively charged ADC throughout the sampling period compared to the WT ADC despite its lower plasma exposure. The $C_{max}$ of total MMAE in the spleen was 2-fold higher for the positively charged ADC, whereas $C_{max}$ of unconjugated MMAE was similar for the two ADCs. Nevertheless, MMAE concentrations declined more rapidly for the positively charged ADC, leading to an overall ~4-fold lower AUC for the payload in the spleen compared to the WT ADC.

Of note, we have previously investigated the PK of positively charged mAbs in mice, and the total mAb PK profiles observed here for both the liver and spleen are not too different from those observed earlier [7].

### 3.6. Tissue-to-Plasma Exposure Ratios

**Figure 7:**
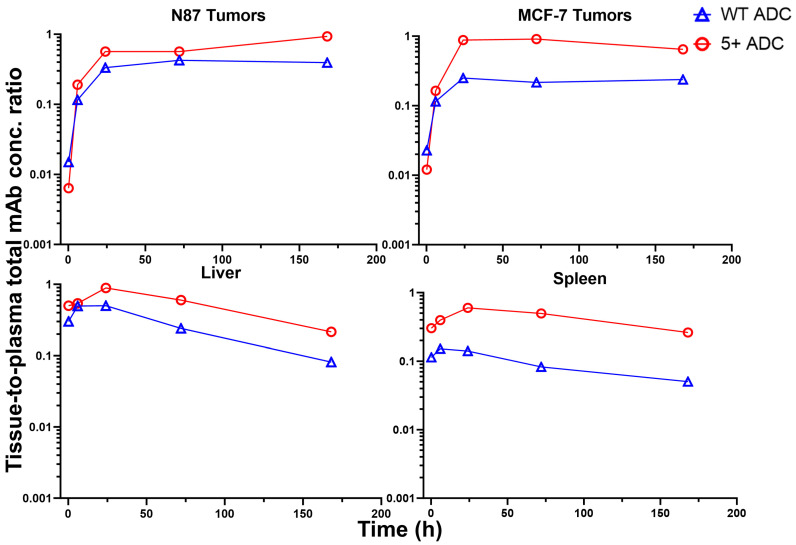
Tissue-to-plasma concentration ratio vs. time profiles for total mAb in N87 tumors, MCF-7 tumors, liver, and spleen for WT and positively charged (+5) ADCs.

**Figure 8:**
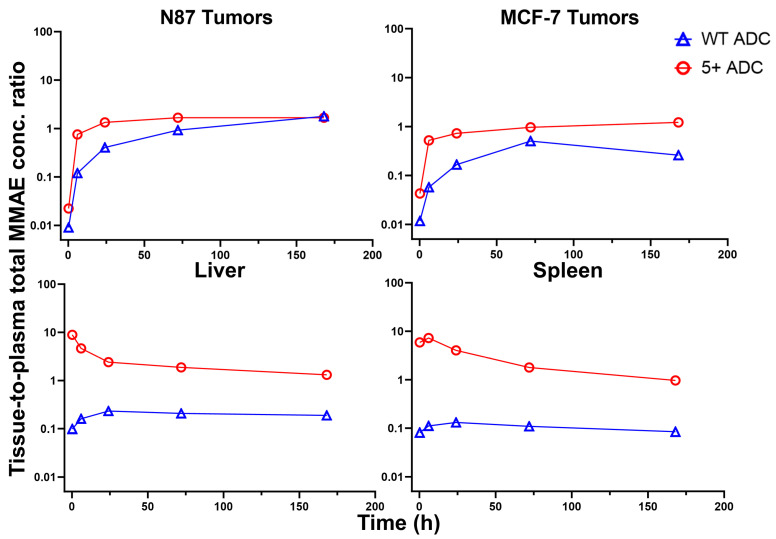
Tissue-to-plasma concentration ratio vs. time profiles for total MMAE in N87 tumors, MCF-7 tumors, liver, and spleen for WT and positively charged (+5) ADCs.

**Figure 9:**
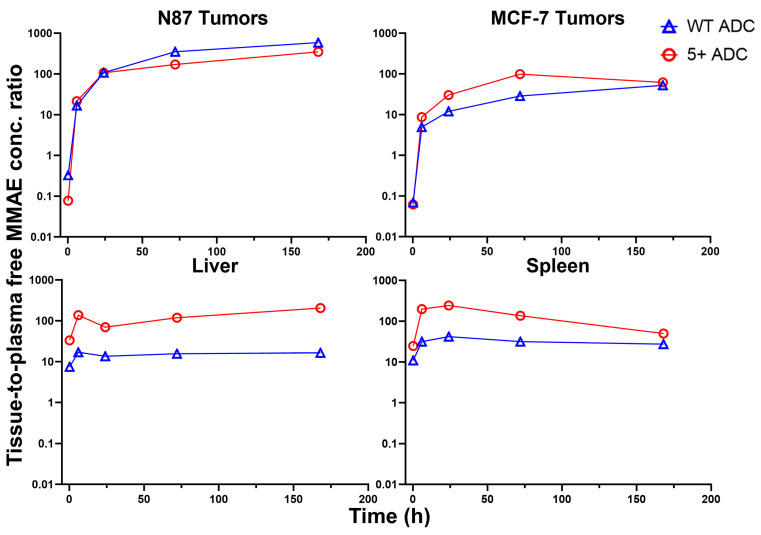
Tissue-to-plasma concentration ratio vs. time profiles for unconjugated MMAE in N87 tumors, MCF-7 tumors, liver, and spleen for WT and positively charged (+5) ADCs.

The tissue-to-plasma concentration ratio vs. time profiles for each ADC analyte are shown in Figure 7 for total mAb, Figure 8 for total MMAE, and Figure 9 for unconjugated MMAE. The tissue-to-plasma AUC_0₋t_ ratios for positively charged and WT ADCs, along with fold differences, are summarized in Table 6.

For total mAb, positively charged ADC demonstrated significantly higher tissue-to-plasma AUC ratios in the liver, spleen, and MCF-7 tumors. However, in NCI-N87 tumors, where HER2 expression is high, the effect of charge on tumor uptake was less pronounced. For total MMAE, the positively charged ADC showed a disproportionate increase in the tissue-to-plasma AUC ratio compared to total mAb, particularly in the spleen and liver. This further supports the hypothesis that higher DAR species preferentially distribute to the liver and spleen. Additionally, the significantly higher tissue-to-plasma $C_{max}$ ratio of total MMAE in the liver and spleen for the positively charged ADC suggests that this effect is more prominent at early time points. For unconjugated MMAE, although overall exposure was lower for the positively charged ADC compared to WT ADC across most tissues, its tissue-to-plasma AUC ratios were higher due to rapid plasma clearance. An exception was observed for free MMAE in NCI-N87 tumors, where tissue-to-plasma ratios were lower for the positively charged ADC, suggesting that HER2 expression may play a role in ADC uptake in high antigen-expressing tumors. In contrast, charge-driven uptake was more evident in tissues lacking a specific targeting site, such as MCF-7 tumors and normal tissues.

### 3.7. In Vivo Efficacy Study and Toxicity Assessment of Positively Charged ADC

**Figure 10:**
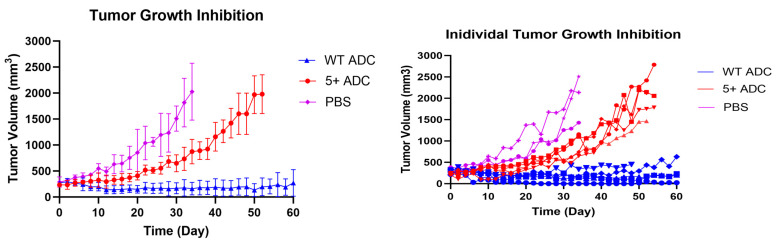
In vivo efficacy of WT and positively charged ADC at 5 mg/kg dose in NCI-N87 tumor-bearing mice. The figure shows the mean tumor growth curves (left) and individual tumor growth curves (right) for each treatment (N = 5) and control group (N = 3).

The tumor growth curves after WT ADC and positively charged ADC administration in NCI-N87-tumor-bearing mice are reported in Figure 10. While complete tumor growth inhibition was observed in mice treated with WT ADC, the positively charged ADC mice group did not show as high efficacy. Even though tumor regression was displayed shortly after dosing, rapid tumor growth was observed 30 days after dosing. Overall, WT ADC showed superior efficacy when compared to the positively charged ADC in NCI-N87 tumors.

**Figure 11:**
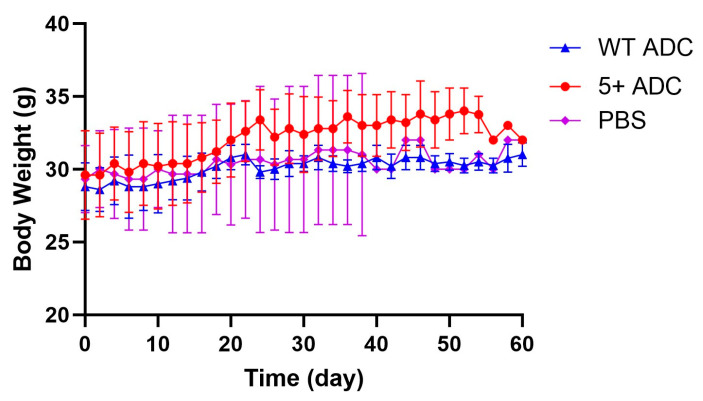
Body weight vs. time profile for NCI-N87 tumor-bearing mice (N = 13) receiving WT ADC, positively charged ADC (each at 5 mg/kg), or control treatment. Mean body weights were recorded over time to assess potential systemic toxicity.

Regarding the toxicity of ADC, the body weight observations from the pilot study were similar between the two ADC and the control group, suggesting no overt toxicity of positively charged ADC in mice at the tested dose of 5 mg/kg (Figure 11).

## 4. Discussion

The emergence of ADCs has paved the way for the development of diverse antibody therapeutics in oncology. Various strategies have been explored to improve the therapeutic index of ADCs, including modifying antibody characteristics, linkers, or payloads [4]. Among these approaches, employing positive charges on the antibody structure has been shown to enhance mAb distribution [6]. Evidence from Stuber’s group has shown a correlation between increasing charges and enhanced tumor exposure of antibodies [8]. Additionally, increasing the positive charge has been reported to expand extravascular distribution volume and promote extravasation into solid tumors [6]. Given that the impact of the antibody-positive charge on ADC potency has been minimally explored, this study aims to investigate the potential of leveraging positively charged antibodies to enhance ADC efficacy through improved tumor exposure.

Positively charged antibodies are believed to exhibit enhanced electrostatic interactions with the negatively charged extracellular matrix (ECM) or the cell membrane, leading to elevated retention in the interstitial space or on the cell membrane [7]. These strengthened interactions at the cell membrane potentially promote the propensity of internalization into endosomes via pinocytosis, resulting in enhanced uptake together with increased intracellular accumulation and clearance of the antibody [7,15]. Therefore, we hypothesized that ADCs generated from these positively charged antibodies would exhibit enhanced tumor accumulation and higher dose potency, leading to an improved therapeutic index of ADCs. We test this hypothesis by generating a positively charged ADC and evaluating its efficacy in vitro and in vivo, together with assessing the biodistribution of different ADC analytes in plasma, tissues, and tumors.

The positively charged ADC was synthesized by conjugating the vc-MMAE linker-payload to an antibody variant with positive charge patches on the variable domain. The positive charge was introduced by mutating residues in the complementarity-determining regions to lysine or arginine. The introduction of a net +5 charge did not significantly alter other physicochemical properties compared to the wild-type trastuzumab. Previous studies have demonstrated that the positively charged variant retained comparable FcRn binding, hydrophobicity, thermal stability, and structural integrity to the wild-type antibody, supporting similarity in developability characteristics [7]. Given the increased pinocytosis of positively charged mAb, one would expect higher in vitro cytotoxicity of positively charged ADC when compared to wild-type ADC. Our cytotoxicity assays showed that when compared to wild-type ADC, the positively charged ADC reduced the IC50 of ADC in low HER2-expressing MCF-7 cells and non-HER2-expressing MDA-MB 468 cells by 13 and 52-fold, respectively, while maintaining the killing effects in high HER2-expressing N87 cells. The increased cytotoxicity observed in vitro suggested higher exposure of positively charged ADC in low-HER2 expressing cells, thus enabling tumor-killing effects of ADC payloads in targets with lower antigen expression. We believe that the enhanced ECM and cell membrane interaction resulting from electrostatic interaction at the membrane interface of positively charged antibodies allows more ADC to be internalized into the endosomes, resulting in an increased fraction of payload being released at the site of action for therapeutic effects.

The enhanced nonspecific pinocytosis of positively charged ADC may also prompt the potential of off-target toxicity in normal tissues, which may subsequently compromise the therapeutic index of ADC. Therefore, we performed biodistribution studies in xenograft models bearing both high HER2-expressing N87 cells and low HER2-expressing MCF-7 cells to investigate the PK characteristics of positively charged ADC. Our results showed that positively charged ADC displayed rapid systemic clearance and enhanced tissue distribution, consistent with previous reports on antibodies with positive charge patches. However, despite similar mAb PK profiles of the two ADCs, we observed higher payload accumulation in non-target tissues, including the spleen and liver, for positively charged ADC, while payload exposure in low antigen-expressing tumors was not as pronounced. Considering the inherent heterogeneity of ADC preparations, we hypothesized that ADC with varying DAR may exhibit different tissue distributions. Specifically, we postulated that higher DAR species could be more prone to accumulation in tissues such as the liver and spleen, and the remaining lower DAR species might distribute to tumors. Moreover, the high tissue-to-plasma ratios observed for unconjugated MMAE are expected, as it is primarily driven by ADC catabolism and MMAE retention within the tissue. Unconjugated MMAE is rapidly cleared from plasma but accumulates in tissues due to its high-affinity binding to tubulin and redistribution to adjacent cells via the bystander effect. Of note, the observed early peak concentration of unconjugated MMAE in mouse plasma aligns with multiple reports documenting similar findings in vc-MMAE ADCs [16,17,18]. This may be attributed to enzymatic cleavage of the Val–Cit linker by plasma proteases such as carboxylesterase 1C [19,20,21,22,23] or to trace amounts of unconjugated MMAE remaining in the ADC formulation, leading to immediate detection upon dosing. Notably, this phenomenon appears to be limited to rodents and is not observed in higher species or humans, as demonstrated by reports of lower Val–Cit linker stability in mouse plasma compared to human plasma [19,20,24]

When examining the PK profiles of ADC analytes in tumors and normal tissues, we noticed that payload exposure was higher at early time points for positively charged ADC with a significantly higher C_max_ when compared to WT ADC in liver and spleen. Given the comparable mAb exposure, this suggests that the elevated payload accumulation in these tissues likely arises from the internalization of high-DAR ADC species. These findings support our hypothesis of the distinct distribution patterns of different DAR species of positively charged ADC. Furthermore, these results highlight the role of not only charge but also hydrophobicity in influencing ADC biodistribution. Indeed, previous studies by Zhao et al. demonstrated the interplay between charge and hydrophobicity of ADCs in driving macropinocytosis-mediated internalization [25]. In agreement with these findings, Lyon’s group suggested an association between increased hydrophobicity of ADC and enhanced hepatic uptake, resulting in accelerated clearance of highly hydrophobic ADC species from the circulation [26]. Their strategy of masking the ADC with a hydrophilic PEG to minimize apparent hydrophobicity successfully reduced the drug clearance and consequently improved ADC potency. Considering the previously reported enrichment of extravasation and vascular permeability of mAb with positive charges [15], we believe that the hydrophobicity-driven liver uptake may become more pronounced for positively charged ADCs. This could lead to a more substantial distribution of high-DAR species to normal tissues compared to targeted tumors. Our study focused on assessing the biodistribution of charged ADC with known average DAR rather than examining individual DAR subpopulations despite hypothesizing that different DAR species may exhibit distinct biodistribution profiles. Further investigations utilizing advanced analytical characterization techniques will be valuable to clarify how charge modifications influence the biodistribution of high-DAR and low-DAR ADC subpopulations, providing deeper insights into the overall impact of charge modifications on ADC efficacy and safety [27].

The discrepancy between in vitro and in vivo PK data prompted us to investigate the in vivo efficacy of positively charged ADCs. Given the reported low accumulation of MMAE payload in tumors, we hypothesized that positively charged ADC might not be as efficacious as the wild-type ADC in a xenograft mouse model bearing high HER2-expressing N87 tumors. Following a single dose of 5 mg/kg of either positively charged ADC, wild-type ADC, or PBS control, we observed significantly greater N87 tumor inhibition in the mice treated with WT ADC than positively charged ADC. While complete tumor regression was achieved in WT ADC-treated animals, tumor growth was rapidly observed in animals administered with positively charged ADC within 20 days. These observations indicate the inferior in vivo efficacy of positively charged ADC when compared to WT ADC in tumor-bearing mice. The limited efficacy observed in positively charged ADC is attributed to inadequate payload exposure at the tumor sites, aligning with our previous hypothesis of different distribution of ADC species with varying DAR. Similar findings were demonstrated by Sun and colleagues in a study investing the DM4-based ADC at different DARs [28]. They also suggested a related trend when higher DAR species displayed faster clearance compared to lower DAR species and thus suffered reduced efficacy in vivo. Similarly, Hamblett et al. reported that despite showing higher potency in vitro, ADC with a DAR of 8 showed comparable efficacy to those with DARs of 2 and 4, likely due to faster clearance and lower systemic exposure observed for higher DAR species in vivo [29]. Thus, in vitro assessment of ADC potency may not be enough to triage ADC molecules for clinical development, and in vivo PK/PD studies in preclinical species should be conducted to further support the clinical development of an ADC.

We also investigated the overt toxicity of positively charged ADC in animals. Monitoring body weight changes throughout the tumor growth inhibition study revealed no significant differences between animals treated with positively charged ADC, WT ADC, or PBS control. Similarly, no skin lesions or local injection site toxicity were observed. However, the PK profiles of positively charged ADC with a high accumulation in highly perfused tissues raised concerns about off-target toxicity. Together with the reduced efficacy in tumor inhibition studies, this possible risk of tissue accumulation would further narrow the therapeutic index of ADC and limit the clinical potential of positively charged ADCs. Moreover, charged mAbs present challenges in achieving the desired DAR during conjugation. While WT ADC with a DAR of 4 was successfully synthesized using a TCEP molar ratio of 3, a relatively higher TCEP equivalent of 8 was required for the positively charged ADC to achieve an acceptable DAR. This difference in TCEP requirement is likely attributed to the intrinsic properties of the mAb. Additionally, positively charged antibodies are generally less stable than the WT due to its charge properties, and the instability and aggregation in our case were minimized using an arginine-PBS storage buffer, which has been shown to enhance solubility and long-term stability of antibodies by improving protein folding and reducing hydrophobic interactions [30,31]. Furthermore, to mitigate variability related to storage and stability, both WT and positively charged ADCs were synthesized and used within a period of one week.

It is worth noting that our study has a few limitations as well. First, our biodistribution analytical methods focused on major organs (plasma, tumors, liver, and spleen) and did not encompass a comprehensive whole-body tissue analysis [12,32]. Second, our in vivo efficacy study was restricted to high-HER-expressing N87 tumors, precluding a direct assessment in tumor sites with low antigen expression. Although our study demonstrates that charged ADCs can influence efficacy in HER2-positive xenograft models, we acknowledge that testing a broader range of tumor types, including HER2-low, HER2-negative, and patient-derived xenograft (PDX) models, is necessary for drawing more comprehensive conclusions. For instance, evaluating charged ADCs in HER2-low or HER2-negative tumors would clarify whether nonspecific pinocytosis meaningfully contributes to tumor accumulation when receptor-mediated internalization is limited. Further investigations will help inform the generalizability of our findings on how charge modifications affect ADC efficacy. Third, the in vivo efficacy study was limited to a single dose. Considering the aim of the study was a proof-of-concept assessment of positively charged ADC, we believed that a representative dose of 5 mg/kg would be sufficient for the initial evaluation of ADC efficacy in vivo. Nonetheless, more dedicated efficacy and safety studies should be conducted to support our conclusions. Finally, our study utilized trastuzumab and its charge variant as the ADC backbone, potentially limiting the generalizability of our findings to ADCs constructed with other antibodies and payloads. More comprehensive studies with broader dose ranges and diverse antibody-payload combinations will be necessary for the generalization of findings suggesting the inferiority of positively charged ADCs. While alternative ADC optimization strategies [33], such as linker modifications [34,35] and DAR optimizations [29], have been widely explored, our study focuses on the physicochemical properties of the antibody backbone, specifically charge and its influence on ADC PK/PD. Future studies could investigate the potential of integrating charge modification with established strategies, such as linker refinement, advanced conjugation methods, or DAR optimization, to further modify ADC efficacy, stability, and safety.

Overall, despite demonstrating promising in vitro activity against both moderate antigen-expressing and low antigen-expressing targets, positively charged ADCs failed to translate their enhanced potency in the in vivo studies. Our findings highlight the interplay between charge and hydrophobicity in influencing ADC distribution and efficacy. Specifically, we hypothesize the tendency of ADC with higher DAR species to favorably accumulate in tissues such as the liver and spleen, and the leftover lower DAR species is delivered to the tumors. This different distribution results in reduced tumor exposure of payload at the target sites and elevated risk of off-target toxicity. Subsequently, we conclude that employing positively charged antibodies to make ADC may not be a viable strategy to improve the therapeutic index of ADCs.

## Figures and Tables

**Table 1:** Parameters for the plasma PK of WT and positively charged (+5) ADCs in mice.

Parameters (Unit)	Positively Charged ADC	WT ADC
Total mAb		
$T_{1/2}$ (h)	77.8	350
CL (mL/h)	0.0449	0.00518
$V_{ss}$ (mL)	4.88	2.57
$C_{max}$ (nM)	716 ± 188	1600 ± 57.4
${AUC}_{0-t}$ (h∙nM)	34,100 ± 5400	113,600 ± 5700
${AUC}_{inf}$ (h∙nM)	44,500	386,000
Total MMAE		
$T_{1/2}$ (h)	41.7	57.5
CL (mL/h)	1.74	0.0452
$V_{ss}$ (mL)	65.2	2.93
$C_{max}$ (nM)	172 ± 38.6	5080 ± 406
${AUC}_{0-t}$ (h∙nM)	2200 ± 473	157,900 ± 7700
${AUC}_{inf}$ (h∙nM)	2300	177,000
Unconjugated MMAE		
$T_{1/2}$ (h)	17.6	70.3
$C_{max}$ (nM)	8.72 ± 2.33	15.8 ± 3.66
${AUC}_{0-t}$ (h∙nM)	23.3	278
${AUC}_{inf}$ (h∙nM)	23.9	331

**Table 2:** PK parameters of WT and positively charged ADCs in NCI-N87 tumors.

Parameters (Unit)	Positively Charged ADC	WT ADC
Total mAb		
$T_{max}$ (h)	24	24
$C_{max}$ (nM)	146 ± 51.6	364 ± 91.5
${AUC}_{all}$ (h∙nM)	22,500	52,600
Total MMAE		
$T_{max}$ (h)	24	72
$C_{max}$ (nM)	45.3 ± 17.6	610 ± 112
${AUC}_{all}$ (h∙nM)	2057	87,300
Unconjugated MMAE		
$T_{max}$ (h)	24	72
$C_{max}$ (nM)	20.1 ± 10.3	403 ± 71.2
${AUC}_{all}$ (h∙nM)	1843	53,106

**Table 3:** PK parameters of WT and positively charged ADCs in MCF-7 tumors.

Parameters (Unit)	Positively Charged ADC	WT ADC
Total mAb		
$T_{max}$ (h)	24	24
$C_{max}$ (nM)	301 ± 107	181 ± 35.1
${AUC}_{all}$ (h∙nM)	28,500	24,000
Total MMAE		
$T_{max}$ (h)	24	72
$C_{max}$ (nM)	28.1 ± 11.2	333 ± 175
${AUC}_{all}$ (h∙nM)	1184	33,550
Unconjugated MMAE		
$T_{max}$ (h)	6	72
$C_{max}$ (nM)	6.52 ± 1.45	36.7 ± 0.613
${AUC}_{all}$ (h∙nM)	519	5148

**Table 4:** PK parameters for WT and positively charged ADCs in the liver.

Parameters (Unit)	Positively Charged ADC	WT ADC
Total mAb		
$T_{max}$ (h)	24	6
$C_{max}$ (nM)	820 ± 300	527 ± 5.61
${AUC}_{all}$ (h∙nM)	42,100	31,300
Total MMAE		
$T_{max}$ (h)	0.167	0.167
$C_{max}$ (nM)	1100 ± 296	502 ± 46.6
${AUC}_{all}$ (h∙nM)	5370	28,700
Unconjugated MMAE		
$T_{max}$ (h)	0.167	0.167
$C_{max}$ (nM)	221 ± 58.1	118 ± 18.9
${AUC}_{all}$ (h∙nM)	1540	3980

**Table 5:** PK parameters for WT and positively charged ADCs in the spleen.

Parameters (Unit)	Positively Charged ADC	WT ADC
Total mAb		
$T_{max}$ (h)	24	0.167
$C_{max}$ (nM)	387 ± 41.8	182 ± 10.5
${AUC}_{all}$ (h∙nM)	26,700	10,800
Total MMAE		
$T_{max}$ (h)	0.167	0.167
$C_{max}$ (nM)	750 ± 164	412 ± 23.3
${AUC}_{all}$ (h∙nM)	4380	16,500
Unconjugated MMAE		
$T_{max}$ (h)	0.167	0.167
$C_{max}$ (nM)	135 ± 41.1	173 ± 24.8
${AUC}_{all}$ (h∙nM)	1970	8520

**Table 6:** Tissue-to-plasma AUC ratios for WT and positively charged ADCs shown as percentages.

Parameters (Unit)	Positively Charged ADC ^a^	WT ADC ^a^
Total mAb		
Liver	123	27.5
Spleen	78.3	9.47
NCI-N87	66.1	46.3
MCF7	83.6	21.2
Total MMAE		
Liver	244	18.2
Spleen	199	10.4
NCI-N87	93.6	55.3
MCF7	53.9	21.3
Unconjugated MMAE		
Liver	6600	1430
Spleen	8440	3070
NCI-N87	7920	19,100
MCF7	2230	1850

^a^ Ratio presented as percentage (%).

## Data Availability

The original contributions presented in this study are included in the article/Supplementary Material. Further inquiries can be directed to the corresponding author.

## Supplementary Materials

The following supporting information can be downloaded at: https://www.mdpi.com/article/10.3390/pharmaceutics17030377/s1, Figure S1. SDS-PAGE analysis of purified positively charged mAb and wild-type (WT) trastuzumab under reducing and non-reducing conditions. Under non-reducing conditions, both positively charged mAb (lanes 2 and 4) and WT mAb (lanes 6 and 8) appear as bands at ~150 kDa. Under reducing conditions, both mAbs (lanes 1 and 3 for positively charged mAb; lanes 5 and 7 for WT mAb) resolve into two bands at ~25 kDa and ~50 kDa, corresponding to the antibody light and heavy chains, respectively; Figure S2. Characteristics of Hydrophobic interaction chromatography (HIC) analysis of WT ADC used for drug–antibody-ratio (DAR) characterization. The previously established HIC protocol [11] was used to determine the DAR of WT ADC. Briefly, mobile phase A contained 1.5 M ammonium sulfate in 50 mM phosphate buffer (pH 7), while mobile phase B consisted of 50 mM phosphate buffer with 20% isopropanol at the same pH. ADCs were analyzed over 18 minutes at a flow rate of 0.8 mL/min, and peak area integration was performed to calculate the overall DAR. Since this protocol was optimized for WT ADC but not for positively charged ADC, an alternative forced deconjugation method was used to estimate the DAR of the latter. WT and positively charged ADCs were spiked into plasma and incubated with cysteine protease papain (Sigma-Aldrich) for 8 hours to achieve complete deconjugation. The total released MMAE concentration was quantified by LC–MS/MS, and the MMAE/internal standard (IS) ratio was recorded. The relative MMAE/IS ratios between WT and positively charged ADCs (~0.7) were used to estimate the DAR of positively charged ADCs. HIC analysis determined an average DAR of around 4 for WT ADC, and the estimated DAR for positively charged ADCs was around 3.
