# Supplementary material for: PK/PD of Positively Charged ADC in Mice
Source: Pharmaceutics. 2025 Mar 17;17(3):377. doi: 10.3390/pharmaceutics17030377 (PMC11944646; doi:10.3390/pharmaceutics17030377)
Supplement: Supplementary file 1 [file pharmaceutics-17-00377-s001.zip › pharmaceutics-3499166-supplementary.pdf]

Supplementary Materials

PK/PD of Positively Charged ADC in Mice

Hsuan-Ping Chang, Huyen Khanh Le, Shufang Liu and Dhaval K. Shah *

Department of Pharmaceutical Sciences, School of Pharmacy and Pharmaceutical Sciences, The State University of New York at Buffalo, Buffalo, NY

* Correspondence: dshah4@buffalo.edu

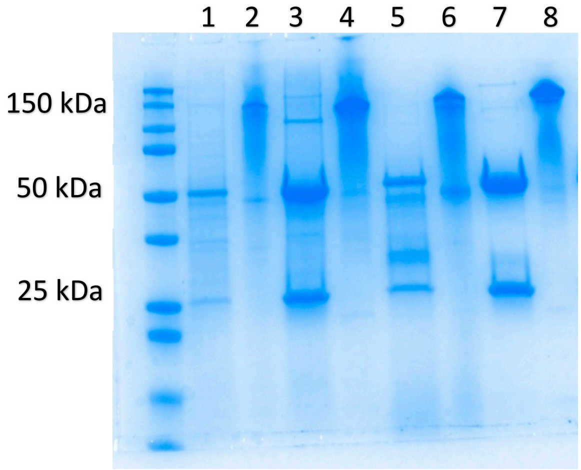

Figure S1. SDS-PAGE analysis of purified positively charged mAb and wild-type (WT) trastuzumab under reducing and non-reducing conditions. Under non-reducing conditions, both positively charged mAb (lanes 2 and 4) and WT mAb (lanes 6 and 8) appear as bands at ~150 kDa. Under reducing conditions, both mAbs (lanes 1 and 3 for positively charged mAb; lanes 5 and 7 for WT mAb) resolve into two bands at ~25 kDa and ~50 kDa, corresponding to the antibody light and heavy chains, respectively.

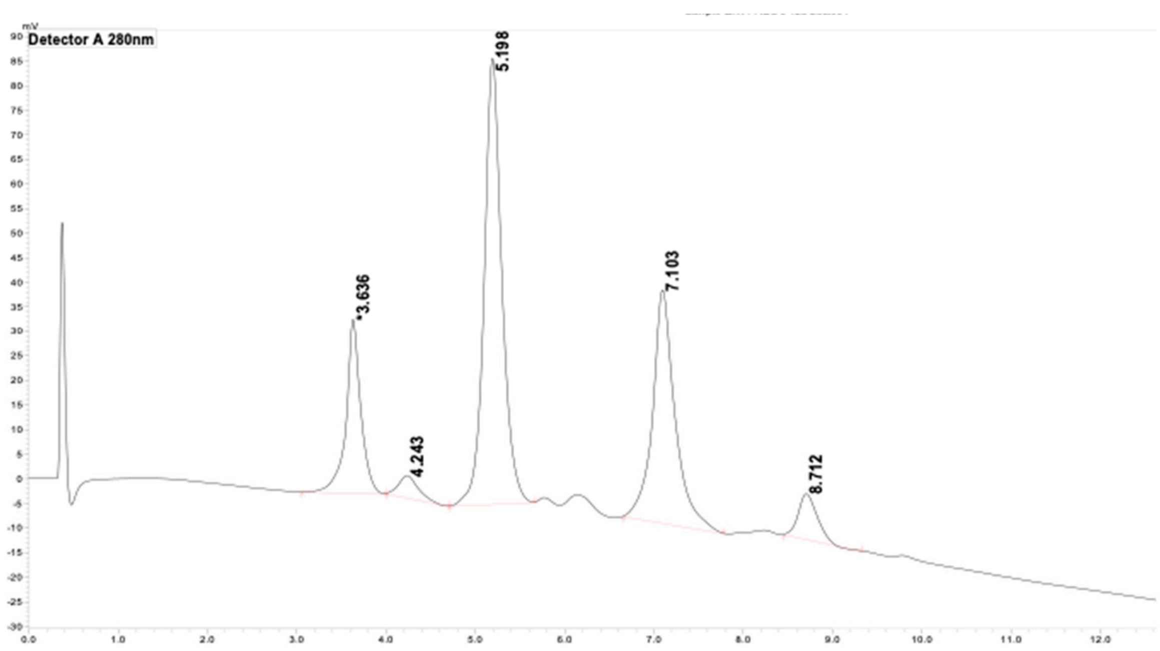

Figure S2: Characteristics of Hydrophobic interaction chromatography (HIC) analysis of WT ADC used for drug-antibody-ratio (DAR) characterization. The previously established HIC protocol [11] was used to determine the DAR of WT ADC. Briefly, mobile phase A contained 1.5 M ammonium sulfate in 50 mM phosphate buffer (pH 7), while mobile phase B consisted of 50 mM phosphate buffer with 20% isopropanol at the same pH. ADCs were analyzed over 18 minutes at a flow rate of 0.8 mL/min, and peak area integration was performed to calculate the overall DAR. Since this protocol was optimized for WT ADC but not for positively charged ADC, an alternative forced deconjugation method was used to estimate the DAR of the latter. WT and positively charged ADCs were spiked into plasma and incubated with cysteine protease papain (Sigma-Aldrich) for 8 hours to achieve complete deconjugation. The total released MMAE concentration was quantified by LC-MS/MS, and the MMAE/internal standard (IS) ratio was recorded. The relative MMAE/IS ratios between WT and positively charged ADCs (~0.7) were used to estimate the DAR of positively charged ADCs. HIC analysis determined an average DAR of around 4 for WT ADC, and the estimated DAR for positively charged ADCs was around 3.
